# RegenDbase: a comparative database of noncoding RNA regulation of tissue regeneration circuits across multiple taxa

**DOI:** 10.1038/s41536-018-0049-0

**Published:** 2018-05-29

**Authors:** Benjamin L. King, Michael C. Rosenstein, Ashley M. Smith, Christina A. Dykeman, Grace A. Smith, Viravuth P. Yin

**Affiliations:** 1 Kathryn Davis Center for Regenerative Biology and Medicine, Mount Desert Island Biological Laboratory, Salisbury Cove, ME 04672 USA; 2 Department of Molecular and Biomedical Sciences, University of Maine, Orono, ME 04469 USA; 3 Graduate School of Biomedical Sciences and Engineering, University of Maine, Orono, ME 04469 USA; 4 University of Maine Honors College, University of Maine, Orono, ME 04469 USA; 5 Present Address: RockStep Solutions, Portland, ME 04101 USA

## Abstract

Regeneration is an endogenous process of tissue repair that culminates in complete restoration of tissue and organ function. While regenerative capacity in mammals is limited to select tissues, lower vertebrates like zebrafish and salamanders are endowed with the capacity to regenerate entire limbs and most adult tissues, including heart muscle. Numerous profiling studies have been conducted using these research models in an effort to identify the genetic circuits that accompany tissue regeneration. Most of these studies, however, are confined to an individual injury model and/or research organism and focused primarily on protein encoding transcripts. Here we describe RegenDbase, a new database with the functionality to compare and contrast gene regulatory pathways within and across tissues and research models. RegenDbase combines pipelines that integrate analysis of noncoding RNAs in combination with protein encoding transcripts. We created RegenDbase with a newly generated comprehensive dataset for adult zebrafish heart regeneration combined with existing microarray and RNA-sequencing studies on multiple injured tissues. In this current release, we detail microRNA–mRNA regulatory circuits and the biological processes these interactions control during the early stages of heart regeneration. Moreover, we identify known and putative novel lncRNAs and identify their potential target genes based on proximity searches. We postulate that these candidate factors underscore robust regenerative capacity in lower vertebrates. RegenDbase provides a systems-level analysis of tissue regeneration genetic circuits across injury and animal models and addresses the growing need to understand how noncoding RNAs influence these changes in gene expression.

## Software: Online tool allows cross-species comparisons of regeneration

A new online resource allows researchers to probe the genomic circuitry that underpins tissue regeneration across a range of injury and animal models. A team led by Benjamin King from the University of Maine in Orono and Viravuth Yin from the MDI Biological Laboratory in Bar Harbor, Maine, USA, created a tool called RegenDbase, the Comparative Models of Regeneration Database. It incorporates gene expression datasets for protein-coding genes and regulatory RNA molecules from zebrafish, axolotl salamanders and mice. As a proof of principle, the researchers used RegenDbase to find RNAs common to heart regeneration in both neonatal mice and zebrafish using an extensive new zebrafish dataset. Twenty-eight new zebrafish regulatory RNA molecules were identified using those data. Future incorporation of datasets from other organisms and human tissues will enable broader cross-species comparisons of regenerative biology.

## Introduction

A limited capacity to repair and regenerate injured and damaged tissues underscores many degenerative and chronic diseases.^1–4^ Regenerative biology seeks to define endogenous and fundamental mechanisms that can be used to stimulate human regenerative capacity. For example, drastically improved outcomes would be realized if necrotic cardiac muscle tissue regenerated after acute myocardial infarction rather than forming scar tissue that reduces cardiac output. Unlike humans, many adult vertebrates, such as ray-finned fishes and urodeles, have the capacity to regenerate many injured tissues. The zebrafish, *Danio rerio*, can regenerate multiple adult tissues including cardiac,^5^ spinal cord,^6^ and fin appendages.^7^ Urodele models of limb regeneration include the axolotl (*Ambystoma mexicanum*)^8^ and newt (*Notophtalmus viridescens*).^9^ Studies of these individual models have advanced our understanding of regeneration genetic circuits and their regulation. Among these mechanisms are microRNAs (miRNAs), small noncoding RNAs that are essential regulators of development and adult tissue regeneration.^10–13^

Comparative studies of multiple models of complex tissue regeneration provide an opportunity to identify signaling pathways that are conserved or unique among models.^14^ Conducting these comparisons within a phylogenetic context also allows for analysis of ancestral and derived traits. These comparative studies pose challenges that existing databases have not resolved, including (1) compiling large profiling datasets on complex tissue regeneration; (2) facilitating comparisons of experiments within and across organisms; and (3) integrating noncoding RNAs as another level of regulatory control.

Current database resources used for analyzing gene expression are limited in terms of breadth of organisms, exclusion of noncoding RNAs, and a lack of focus on biological processes. The Limbform resource integrated 829 experiments to predict how temporal and spatial patterns of gene expression are associated with anatomical structures during appendage regeneration.^15^ While eight vertebrate species are represented in Limbform, zebrafish and mice are not among them. miRNAs, potent regulators of regeneration, are not represented in this resource. The Regeneration Gene Database (REGene) provides information about 948 genes gathered from the literature using Gene Ontology (GO) annotations and abstracts, but does not present large-scale gene expression datasets from regenerating tissues.^16^ Existing gene expression resources such as the Gene Expression Omnibus,^17^ Expression Atlas,^18^ or Genotype-Tissue Expression Project,^19^ while informative, do not specifically focus on regeneration or robustly support comparisons across datasets. Analyses of miRNA and mRNA expression data along with miRNA target predictions are well represented for 73 human datasets in miRGator.^20^ However, miRGator lacks the focus on tissue repair and regeneration. A database resource that aims to provide a systems-level analysis of tissue regeneration genetic circuits would ideally include these existing resources and provide complementary functionality pertinent to comparative studies of regeneration.

In this work, we describe a newly created Comparative Models of Regeneration Database (RegenDbase; https://regenDbase.org), which focuses on understanding the regulatory control of noncoding RNAs on evolutionarily conserved regeneration signaling pathways. Employing the unique functionality and web interface of RegenDbase, we studied newly generated zebrafish heart regeneration miRNA and mRNA expression datasets and made comparisons with similar data from neonatal mouse heart regeneration. RegenDbase was used to make predictions about stage-specific interactions between miRNAs and mRNAs during zebrafish heart regeneration. We extended our understanding of noncoding RNAs through the identification of long noncoding RNA (lncRNAs) and their putative target transcripts. We characterized a subset of six previously annotated and five novel, differentially expressed lncRNAs that were located antisense or adjacent to differentially expressed protein-coding genes. Finally, we compared early stages of zebrafish and neonatal mouse heart regeneration to identify orthologous genes that were commonly differentially expressed. The integrated functionality within RegenDbase has the potential to advance our understanding of tissue regeneration by accelerating the identification of candidate genes for functional studies through a cross-species comparative strategy.

## RESULTS

### Comparative Models of Regeneration Database

**Fig. 1:**
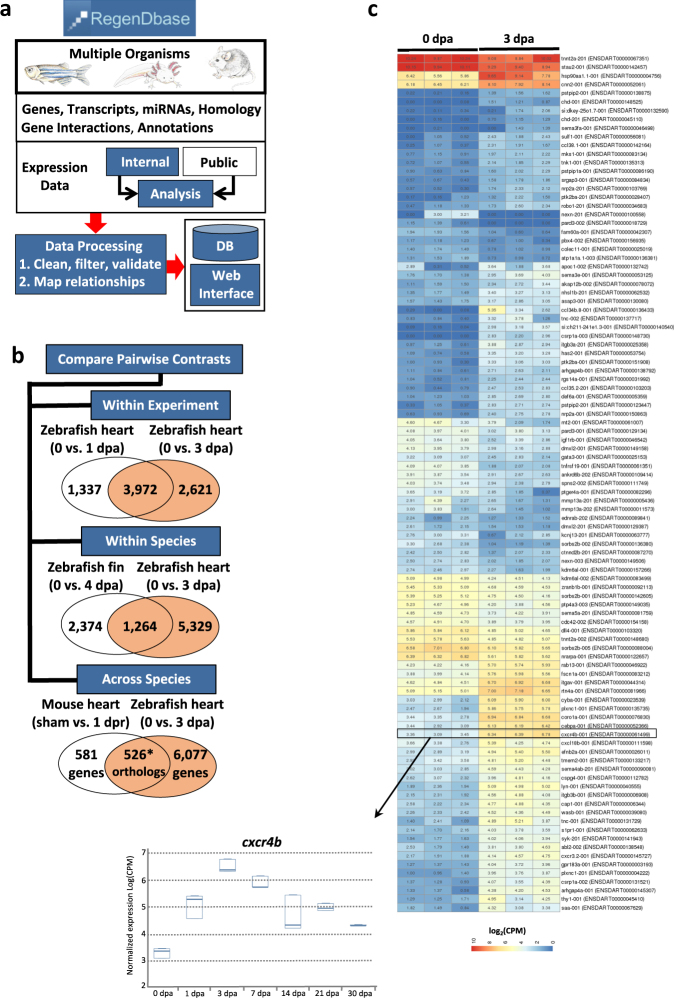
RegenDbase provides fundamental analyses of regeneration circuits within individual experiments and comparative analyses across experiments and species. **a** Organizational workflow for multi-layered data processing and analysis of published (public) and unpublished (internal) gene expression datasets. **b** Examples of how RegenDbase was used to compare: (i) genes common or unique to two pairwise contrasts of early stages of zebrafish heart regeneration (0 vs. 1 dpa and 0 vs. 3 dpa); (ii) genes common or unique to contrasts from early stages of zebrafish caudal fin regeneration (0 vs. 4 dpa) and zebrafish heart regeneration (0 vs. 3 dpa); (iii) comparative analyses of early zebrafish heart (0 vs. 3 dpa) and neonatal mouse heart (0 vs. 1 dpa) regeneration showing the number of orthologs commonly differentially expressed along with the number of zebrafish and mouse genes unique to each model. **c** Heatmap representing genes from precomputed pairwise contrasts from a subset of significantly up- and downregulated transcripts between uninjured and 3 days post-amputation (dpa) regenerating adult ventricles. The temporal expression profile for *cxcr4b*, an essential regeneration gene, is shown as a boxplot in the web interface

The Comparative Models of Regeneration Database (RegenDbase) is a resource designed to identify and compare signaling pathways that underlie tissue regeneration across research models (Fig. 1). RegenDbase is an integrated, multi-faceted platform consisting of data analysis pipelines, a relational database, database load software, and web interface software. Represented within RegenDbase are genes, transcripts, and miRNAs from Ensembl^21^ and miRBase^22^ for zebrafish, axolotl, and mouse. In order to integrate additional studies in the future, we also represent human and *C. elegans* genes, transcripts and miRNAs. GO^23^ functional annotations, pathway assignment from BioSystems,^24^ and homology relationships among genes from OrthoDB^25^ are also integrated together in the RegenDbase platform (Fig. 1a; Supplementary Fig. S1).

Integration of published and unpublished data derived from several disparate sources required defined rules to clean and validate data prior to mapping relationships. Gene and miRNA expression data, currently from zebrafish, axolotl, and mouse (Supplementary Table S1), were reanalyzed using consistent microarray or RNA-Seq analysis workflows to incorporate up-to-date gene annotation. Analysis results were represented as pairwise contrasts between pairs of sample groups and can be filtered by several different strategies including significance, direction of fold change, GO annotations, and BioSystems pathway membership.

A key feature of RegenDbase is the methodology to define the regulatory control of noncoding RNAs during regeneration. First, RegenDbase enables investigation of how miRNAs regulate differentially expressed genes in two ways. RegenDbase generates miRNA target predictions for human, mouse, zebrafish, and *C. elegans* using up-to-date Ensembl annotation of 3p UTR sequences. Using these miRNA target predictions, differentially expressed genes may be filtered by whether they are predicted targets of any miRNAs specified in a list (Fig. 1a). Alternatively, differentially expressed genes may be identified that are co-targeted by up to three miRNAs. Second, RegenDbase can identify differentially expressed lncRNAs and associated neighboring genes along the chromosome that represent putative lncRNA target genes. Differentially expressed genes may be filtered by proximity to identify gene pairs that are adjacent to one another within a window size up to 10 genes. These gene pairs may represent potential cases where an lncRNA could regulate a protein-coding gene^26^ or gene clusters.^27^

RegenDbase provides a unique approach for comparing gene and miRNA expression data. Filtered subsets of differentially expressed mRNAs or miRNAs from two pairwise contrasts may be compared to identify shared or unique genes (Fig. 1b, c). These comparisons may be applied within and across experiments and organisms. For instance, comparative analyses from a single experiment can be used to survey different stages of regeneration and identify candidate markers unique to a stage, or common across multiple stages. By comparing uninjured and 3 days post-amputation (dpa) time points of heart regeneration, we can readily identify known regeneration genes, such as the chemokine (C-X-C motif) receptor 4b, *cxcr4b*,^28^ or novel candidate genes for functional studies (Fig. 1c). RegenDbase has the functionality to rapidly display the expression changes for *cxcr4b* or any other gene across all stages of heart regeneration (Fig. 1c). Alternatively, intra-species comparisons may be performed to identify shared genes for regeneration following different injury models. For example, the zebrafish heart ventricular resection injury may be compared to the cryoinjury model. Alternatively, the expression of proliferative genes during blastema formation during regeneration of zebrafish caudal fins could be compared to early stages of zebrafish heart regeneration. Integration of homology data into RegenDbase permits searches of orthologous genes across regeneration datasets. This functionality allows for comparisons across species, such as comparing zebrafish and neonatal mouse heart regeneration (Fig. 1a).

### Integrating a new dataset for zebrafish heart regeneration

To better understand the transcriptional changes in gene expression during zebrafish heart regeneration, we profiled miRNA and mRNA expression across a time course of zebrafish heart regeneration, from 0, 1, 3, 7, 14, 21 to 30 dpa. These time points encompass the various cellular processes of heart regeneration that are coordinated to ensure synthesis of new heart tissue and restoration of tissue function.^29^ These stages include clotting and endocardial activation (1 dpa), epicardial activation and cardiomyocyte dedifferentiation and proliferation (3 dpa), peak cardiomyocyte proliferation (7 dpa), vascularization and muscle integration with the uninjured heart tissue (14 dpa), electrical coupling and clot dissolution (21 dpa), and functional recovery of tissue function (30 dpa). We compared the uninjured (0 dpa) samples to each of the subsequent stages and integrated these pairwise contrasts into RegenDbase using both noncoding and protein encoding transcripts.

### Stage-specific miRNA expression profiles of zebrafish heart regeneration

**Fig. 2:**
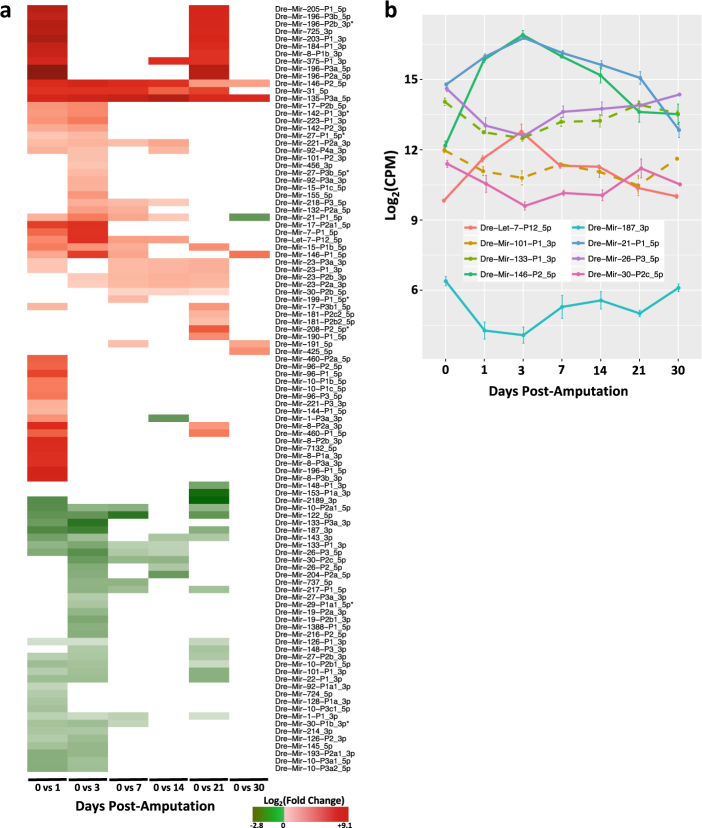
miRNAs are differentially regulated during zebrafish heart regeneration. **a** Heatmap of differentially expressed miRNAs for each pairwise comparison between uninjured (0 dpa) and subsequent time points. Mature miRNA nomenclature is from miRGeneDB. **b** Expression profile of miRNAs previously demonstrated to be required for zebrafish heart regeneration (Dre-Mir-133-P1_3p and Dre-Mir-101-P1_3p), miRNAs that were upregulated (Dre-Mir-21-P1_5p, Dre-Mir-146-P2_5p and Dre-Let-7-P12_5p), and miRNAs that were downregulated (Dre-Mir-26-P3_5p, Dre-Mir-30-P2c_5p, and Dre-Mir-187_3p) during early stages (1 and 3 dpa) of heart regeneration. Error bars indicate standard error of the mean

miRNAs are critical regulators of gene expression during development and regeneration.^10,11,13,14,30,31^ However, a comprehensive atlas of miRNA expression throughout heart regeneration has not been previously reported. Our analyses of zebrafish heart regeneration identified a total of 103 mature miRNAs that were differentially expressed between 0 dpa and at least one stage during heart regeneration (Fig. 2a; Supplementary Table S2). A total of 38 (37%) miRNAs were differentially expressed at a single time point including 18 at 1 dpa, 12 at 3 dpa, 1 (Mir-199-P1_5p*) at 7 dpa, 6 at 21 dpa, and 1 (MiR-425_5p) at 30 dpa. Only Mir-135-P3a and Mir-146-P2, an miRNA upregulated in several cancers,^32^ were consistently upregulated across all time points. This general pattern of unique and common miRNAs suggests that there are stage-specific and overlapping regulatory networks.

Previous regeneration studies from our group and others have shown that the magnitude and volume of gene expression changes are most robust at early time points following tissue injury.^10,11,14,33^ Consistent with these observations, we noted that 1 and 3 dpa time points had more differentially expressed mature miRNAs compared to 0 dpa than later time points. Thirty-six miRNAs were commonly differentially expressed at both 1 and 3 dpa with 35 miRNAs unique to 1 dpa and 22 unique to 3 dpa. The known regulator of zebrafish heart regeneration, Mir-133-P3a_3p,^12^ was one of 36 commonly downregulated miRNAs at 1 and 3 dpa (Fig. 2b). During peak cardiomyocyte proliferation at 7 dpa, 23 of the 26 differentially expressed miRNAs are conserved with humans and associated with the regulation of cellular proliferation and cancer, such as Mir-21-P1_5p (ref. ^34^) (Fig. 2b). Eight of the downregulated miRNAs conserved with humans (Mir-1-P1_3p, Mir-10-P2a1_5p, Mir-26-P3_5p, Mir-30-P1b_3p, Mir-30-P2c_5p, Mir-122_5p, Mir-133-P1_3p, Mir-217-P1_5p) were tumor suppressors downregulated in cancer.^35,36^ Of the 17 upregulated miRNAs, 9 (Mir-21-P1_5p, Mir-23-P1_3p, Mir-23-P3a_3p, Mir-30-P2b_5p, Mir-31_5p, Mir-132-P2a_5p, Mir-135-P3a_5p, Mir-191_5p, Mir-221-P2a_3p) have been positively associated with cancer, but 8 (Let-7-P12_5p, Mir-15-P1b_5p, Mir-23-P2a_3p, Mir-23-P2b_3p, Mir-146-P1_5p, Mir-146-P2_5p, Mir-199-P1_5p*, Mir-218-P3_5p) have been described as tumor suppressors.^35–37^ The 21 dpa time point had the third largest set of differentially expressed miRNAs, and these overlapped more with 1 dpa (30 miRNAs) and 3 dpa (18 miRNAs) than later time points of 14 dpa (10 miRNAs) and 30 dpa (2 miRNAs). Furthermore, the common subset of miRNAs shared between 1 and 3 dpa were consistently up- (16 miRNAs) or downregulated (20 miRNAs). Among the downregulated miRNAs at 1, 3, and 21 dpa was the known regulator of zebrafish heart regeneration, Mir-101-P1_3p (ref. ^13^) (Fig. 2b). The stage-specific modulations in miRNA expression suggest that subsets of miRNAs regulate distinct cellular processes during heart regeneration.

### mRNA expression profiling of zebrafish heart regeneration

Comprehensive mRNA expression profiling studies provide an unbiased and invaluable gene catalog for defining the genetic circuits that orchestrate heart regeneration. Consistent with the miRNA expression profiles, subsets of genes were temporally activated and repressed, as shown by sorting genes by the average level of normalized expression across samples at each time point (Supplementary Fig. S2, Supplementary Table S3). Pairwise contrasts between each injury time point with uninjured tissues showed that early stages of regeneration (1 and 3 dpa) had the largest number of differentially expressed genes (Supplementary Table S4). One dpa had 3005 genes and 3 dpa had 3533 genes with 2225 genes in common. Comparisons with subsequent time points had fewer differentially expressed genes: 1178 at 7 dpa, 1065 at 14 dpa, 653 at 21 dpa, and 882 at 30 dpa.

These differentially expressed genes were assigned to annotated cellular processes and shown as a percentage of genes associated with metabolism, heart development, immune function, cell migration, and cell proliferation (Supplementary Fig. S2). These diverse cellular responses illustrate the dynamics of biological processes during regeneration. The proportion of immune, cell migration, and cell proliferation genes was relatively constant. However, the proportion of metabolism, development, and heart development genes had large changes. For metabolism genes, both the smallest proportion of upregulated and largest proportion of downregulated genes were found at 7 dpa. The increase in proportions of developmental process and heart development genes peaks at 21 dpa before returning to baseline. Looking across all pairwise-stage comparisons, we identified 11 genes that were consistently upregulated and 103 that were consistently downregulated (Supplementary Table S5). Two examples of consistently upregulated genes include the suppressor of cytokine signaling 3a (*socs3a*) and myelocytomatosis oncogene b (*mycb*).

Our analyses identified 43 genes that were differentially expressed and previously shown to be associated with zebrafish heart regeneration (Supplementary Table S6). These include upregulation of retinoic acid (RA) signaling and the RA synthesis enzyme, aldehyde dehydrogenase 1 family, member A2 (*aldh1a2*). The RA signaling cascade is rapidly activated in the endocardium and epicardium to create an instructive microenvironment for cardiomyocyte proliferation.^38^ Fibronectin 1a and 1b (*fn1a* and *fn1b*), Wilms tumor 1b (*wt1b*), and the *tcf21* and *tbx18* transcription factors were highly upregulated by 1 and 3 dpa, consistent with essential roles in epicardial activation.^39–41^ Cardiomyocyte migration requires expression of the chemokine, *cxcl12b*, and its receptor, *cxcr4b*.^28^ Both transcripts were upregulated during early stages of regeneration (Fig. 1c). Two of the genes, *cx43* and *myl7*, have been associated with heart regeneration. *cx43* is required for heart regeneration and has been shown to be regulated by miR-133a.^12^ The cardiomyocyte myosin light chain 7 gene, *myl7*,^42^ is a commonly used marker to identify differentiated cardiomyocytes.

In addition to the identification of known heart regeneration genes, we identified promising new candidate genes that were also differentially expressed and had known association with heart development. Within this group of 114 genes, we identified a subset of 19 genes that also had known roles in cellular migration (Supplementary Table S5). The RAS oncogene member, *rap1b*, a small GTPase that functions with fibronectin (*fn1*) to regulate integrin signaling,^43^ was highly upregulated at 1, 3, and 14 dpa. Zebrafish *rap1b* morphants displayed myocardium defects and impaired heart contractility.^44^ Similarly, the cysteine and glycine rich protein 1a, *csrp1a*, was upregulated at 1, 3, 7, and 14 dpa. *csrp1a* regulates mesoderm cell migration through the noncanonical Wnt pathway during heart development.^45^ Given that depletion of *rap1b* and *csrp1a* activity culminates in heart defects and embryonic lethality,^44,45^ defining potential roles during heart regeneration will require inducible strategies to deplete gene function in the adult animal.

Our analyses of the 114 differentially expressed genes also identified natriuretic peptide receptor, *npr3*, and Semaphorin factor, *sema3d*, genes with roles in heart development and cellular proliferation (Supplementary Table S7). The natriuretic peptide receptor, *npr3*, regulates cardiomyocyte proliferation under conditions of low levels of natriuretic peptides, *nppa* and *nppb*.^46^ Semaphorins are secreted factors that have been shown to have numerous roles in development of the nervous system as well as the neural crest. Morpholino knockdown of *sema3d* during embryonic development showed defective migration of neural crest cells to the primary heart field prior to heart tube formation.^47^ Lack of cell migration resulted in 20% fewer cardiomyocytes in the primary heart field. Based on their expression profiles and known function in heart development, *npr3* and *sema3d* are strong candidate genes for functional studies in heart regeneration.

Two genes demonstrate the dynamics of gene expression during heart regeneration as they were upregulated at early time points and then downregulated later, or vice versa. The extracellular matrix gene, transgelin (*tagln*), is a highly conserved gene expressed in smooth muscle as well as skeletal and cardiac muscle during mouse development.^48^ *Tagln* was upregulated at 1, 3, 7, and 14 dpa, and then downregulated at 21 and 30 dpa (Supplementary Fig. S3). The transducer of ERBB2 (*tob1a*) is required for dorsoventral patterning and inhibits proliferation by blocking β-catenin/LEF-1 transcriptional complexes.^49^ Consistent with its negative regulation of proliferation, *tob1a* was downregulated by 2.04-fold at 1 dpa and 2.71-fold at 3 dpa and then upregulated by 1.82-fold at 21 dpa (Supplementary Fig. S3). These two genes represent novel candidates for heart regeneration based on their expression patterns and documented roles during development.

### Stage-specific clustering of biological processes during zebrafish heart regeneration

**Fig. 3:**
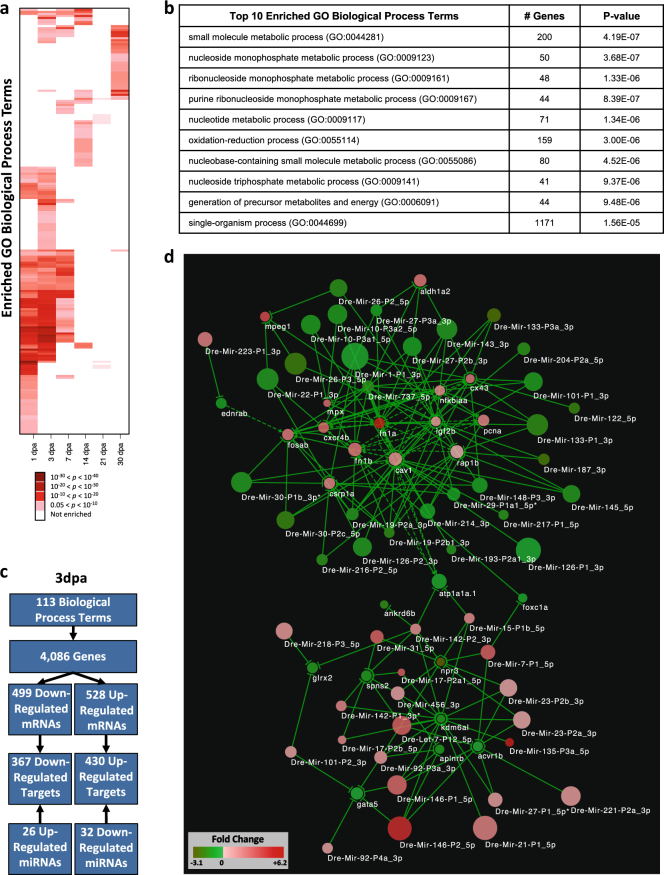
Regulation of enriched Gene Ontology (GO) biological process terms by miRNAs. **a** Patterns of enriched GO terms for differentially expressed mRNAs at 1, 3, 7, 14, 21, and 30 dpa relative to 0 dpa, as shown by two-dimensional hierarchical clustering. Enriched terms are colored from white (not enriched) to red (enriched) based on log_2_(FDR). **b** Top 10 enriched GO terms for genes differentially expressed at 3 dpa relative to 0 dpa. **c** Workflow to identify set of mRNAs differentially expressed at 3 dpa relative to 0 dpa predicted to be targeted by miRNAs that were also differentially expressed at 3 dpa relative to 0 dpa. **d** String analysis depicting a subset of miRNA–mRNA network for 3 dpa relative to 0 dpa containing 22 upregulated and 29 downregulated miRNAs predicted to regulate 14 upregulated and 11 downregulated mRNAs known to be required for zebrafish heart regeneration

To better understand the dynamic regulation of diverse biological processes during heart regeneration, we computed enriched GO Biological Process terms for each stage using GOrilla.^50^ Across all stages, a total of 185 unique Biological Process terms were enriched. Like the pattern of differentially expressed miRNAs, Fig. 3 shows sets of enriched GO terms that specifically define each stage of regeneration. One hundred and nine processes define 1 dpa, 112 for 3 dpa, 43 for 7 dpa, 18 for 14 dpa, 11 for 21 dpa, and 30 for 30 dpa (Supplementary Table S8). The top 10 enriched GO terms at 3 dpa are listed in Fig. 3b. Interestingly, the vast majority of these biological processes are annotated to metabolic processes. A subset of GO processes at an individual stage overlapped with neighboring stages, with 73 enriched processes shared between 1 and 3 dpa, 39 between 3 and 7 dpa, 2 between 7 and 14 dpa, and 2 between 14 and 21 dpa. There was no overlap between 21 and 30 dpa. The overlap of 73 GO terms between 1 and 3 dpa have parental terms including Nucleotide Biosynthesis, Response to Stimulus, Protein Folding, Actin Cytoskeleton Organization, and Generation of Precursor Metabolites and Energy.

The Immune System Process GO term was enriched at 1 and 7 dpa. At the individual gene level, a total of 211 Immune System Process genes were differentially expressed for at least one of the time points. Approximately 18% of the 211 Immune-Related Genes were consistently differentially expressed at 1, 3, and 7 dpa. *Epor, clpxa, slc11a2*, and *zgc:101846* were consistently downregulated across all time points, and *epor*,^51^ *clpxa*,^52^ and *slc11a2*^53^ have known roles in hematopoiesis or erythropoiesis. How and to what extent these immune genes influence tissue regeneration is less understood.

Although tissue regeneration is generally regarded as a continuum of multiple cellular processes that coordinate the replacement of dying tissue with functional cells, we noted that 88 (47%) of the 185 enriched processes were specifically enriched at a single time point, suggesting that these processes align with distinct stages. The time point with the most stage-specific terms was 1 dpa, with 31 unique enriched processes. As a percentage, 14, 21, and 30 dpa had the highest proportions of stage-specific terms, 39% (7/18), 45% (5/11), and 77% (23/30), respectively. For example, Negative Regulation of Cell Fate Commitment (GO:0010454) was enriched at 14 dpa (*p* = 0.00085) with four differentially expressed genes, *fgf17, klf2a, klf2b*, and *tmem88a* (Supplemental Table S8). Both Krupple-like transcription factors, *klf2a* and *klf2b*, were upregulated at 14 and 21 dpa, and are associated with pluripotency and self-renewal of mammalian embryonic stem cells.^54^ Studies of zebrafish development have demonstrated essential roles for *klf2a* and *klf2b* in ectoderm development.^55^

### miRNA control of regenerative processes

After defining a high-level map of enriched Biological Process terms for each stage, we next asked which miRNAs regulate biological processes involved with early events in heart regeneration. Cardiomyocyte proliferation and migration are required for regeneration; these coupled processes begin by 1 dpa and peak between 3 and 7 dpa.^56^ Using expression data from 3 dpa, we identified the subset of predicted target genes for the 26 significantly upregulated and 32 downregulated miRNAs that were annotated to any of the 112 enriched GO Biological Process terms (Fig. 3c). As miRNA binding to target gene can result in mRNA degradation,^57^ we required that target genes have inverse expression compared to the miRNA. For upregulated miRNAs, there were 367 downregulated predicted targets and 430 upregulated predicted targets of the downregulated miRNAs. Among these targets were 14 upregulated and 11 downregulated genes previously identified to be required for heart regeneration and/or development (Fig. 3d). A general feature of this network is that several miRNAs are predicted to regulate the same target genes, suggesting synergistic downregulation of the mRNA. This network includes the known miRNA targeting interaction between the downregulated miRNA, Mir-133-P3a_3p, and the upregulated target cell junction gene, connexin 43 (*cx43*)^12^ (Fig. 3d).

Recently, our group demonstrated that miR-101 depletion was required for zebrafish heart regeneration by stimulating expression of the transcription factor and miR-101 target gene, *fos*.^13^ To further explore potential roles in which miR-101 may regulate transcription factors, we used RegenDbase to identify all transcriptional factors that were upregulated at 3 dpa and predicted to be targeted by miR-101. Six transcription factors were identified, including two members of the FOS family, *fos* and *fosl2* (Supplementary Table S9). The macrophage and B-cell transcription factors, *spi1a* and *spi1b*, are both upregulated. The C/EBP transcription factor, *cebpb*, has a required role in epicardial activation during zebrafish heart development and following injury.^58^ This analysis shows the utility of RegenDbase to identify both known and new candidate genes through the integration of miRNA targeting information. Although we did not consider multiple miRNAs in this analysis, the RegenDbase miRNA search function can be used to identify genes co-targeted by two or three miRNAs. Thus, RegenDbase is a powerful tool to elucidate miRNA regulatory networks in regeneration.

### lncRNA expression during zebrafish heart regeneration

lncRNAs are multi-exonic transcripts >200nt without known protein function that control gene expression at the epigenetic, transcription and post-transcriptional levels.^59,60^ Recently, lncRNAs have been shown to control key genetic circuits important for development, disease, and tissue repair.^61–66^ The identification and extent that lncRNAs control cardiac regeneration circuits, however, remain largely unknown. Employing the RegenDbase functionality for analyses of RNA-sequencing dataset across zebrafish heart regeneration, we identified a subset of previously annotated lncRNAs that were differentially expressed during 1 and 3 dpa of regeneration and the antisense or adjacent protein-coding genes that were also differentially expressed (Supplementary Fig. S1).

**Fig. 4:**
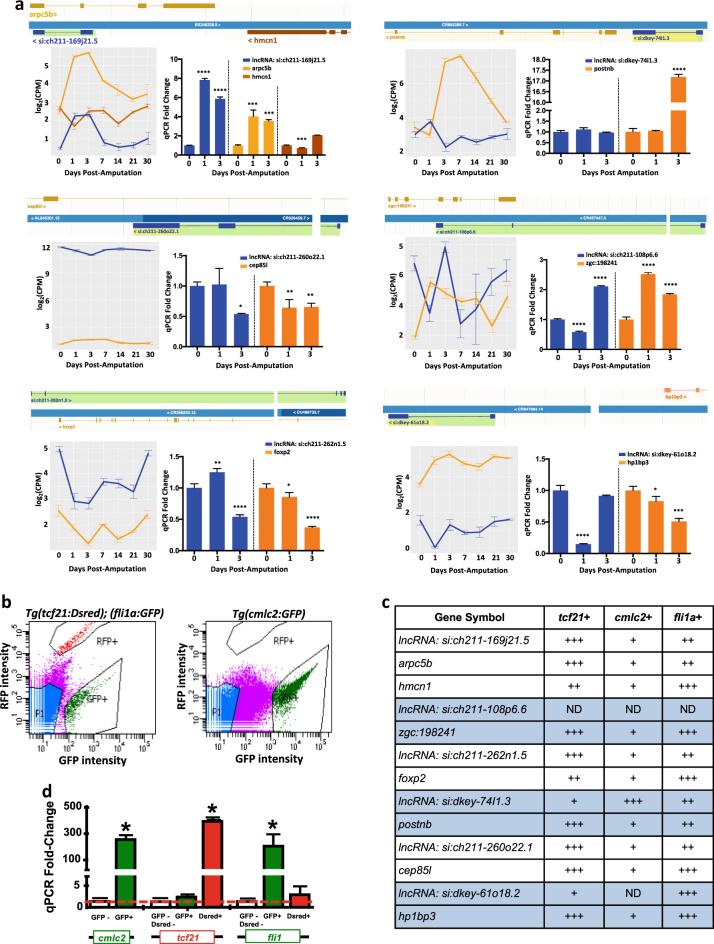
Identification of known lncRNAs that were differentially expressed during early stages of zebrafish heart regeneration. **a** Schematic showing relative genomic location of lncRNAs and corresponding protein-coding genes from Ensembl, gene expression levels from RNA-Seq in 0, 1, 3, 7, 14, 21, and 30 dpa, and qPCR validation of expression in 0, 1, and 3 dpa regenerating ventricles. Error bars indicate standard error of the mean (s.e.m). **b** Flow cytometry plots showing gating of epicardial *(Tg(tcf21:Dsred))*, endocardial *(Tg(fli1a:GFP))*, and cardiomyocyte *(Tg(cmlc2:GFP))* cells from 3 dpa dissociated ventricles. **c** Relative expression of lncRNAs and antisense or adjacent protein-coding gene in *tcf21+*, *cmlc2+*, and *fli1+* cells at 3 dpa by qPCR studies (*ND* not detected, *(+)* low expression, *(++)* 1.5–2.0-fold increase, *(+++)* 2.0 or higher-fold increase in expression). Error bars indicate s.e.m

Differentially expressed lncRNAs were first identified by annotated biotype. As some lncRNAs have been shown to regulate the expression of neighboring protein-coding genes, we used the RegenDbase proximity search features to identify the subset of lncRNAs that have overlapping gene coordinates or that were adjacent to a differentially expressed protein-coding gene. *Lnc-si:ch211-169j21.5* transcript is antisense to *arcp5b*, encoding a tumor suppressor, and adjacent to the extracellular matrix gene, hemicentin 1 (*hmcn1*). Expression of *hmcn1* is inversely correlated with *lnc-si:ch211-169j21.5* at 1 and 3 dpa (Fig. 4a). Mouse *hmcn1* has been shown to have a role in myocardium remodeling in a mouse model of myocardial infarction.^67^ *Lnc-si:ch211-260o22.1* also had an opposite expression pattern to the centrosomal protein 85 like (*cep85l*) gene. Translocations involving human *CEB85L* have been found in patients with myeloproliferative neoplasms.^68^ The *lnc*-*si:ch211-260n1.5* spans several exons of the *foxp2* transcription factor on the opposite strand. Mouse *foxp2* mutants have abnormal electrical conduction systems, displaying R-wave amplitudes.^69^ The growth factor periostin (*postnb*) was upregulated and the neighboring *lnc-si:dkey-74i1.3* was downregulated. The *lnc-si:ch211-108p6.6* had opposite expression to *zgc:198241*, a gene that encodes a protein with an immunoglobulin domain. A regulator for cell cycle progression, *hp1bp3*,^70^ was also upregulated and its neighboring *lnc*-*si:dkey-61o18.2* was downregulated. The expression changes for lncRNAs and potential target transcripts were validated using qPCR analysis from uninjured, 1, and 3 dpa regenerating heart tissues (Fig. 4a). Expression by qPCR correlated with the RNA-Sequencing results for all lncRNAs except for *lnc-si:ch211-262n1.5* and *lnc-si:dkey-74i1.3* at 1 dpa. The expression patterns for the protein-coding genes were also confirmed by qPCR except for *hmcn1, ceb85l, foxp2* at 1 dpa and *hp1bp3* (Fig. 4a).

Next, we examined the cellular distribution of these six lncRNAs and neighboring protein-coding genes at 3 dpa within the three major cardiac tissues: epicardium, myocardium, and endocardium. We resected adult zebrafish hearts, dissociated ventricles, and isolated cells using fluorescence-activated cell sorting (FACS) (Fig. 4b, c). Total RNA was subsequently extracted for qPCR studies. Interestingly, these studies showed that most lncRNAs and their putative target mRNAs are enriched in the epicardium and endocardium, two tissues that craft an instructive microenvironment for new cardiomyocyte synthesis (Fig. 4c). We observed strong expression of *arpc5b, hmcn1, zgc:198241, foxp2, postnb*, and *cep85l* in the epicardium and endocardium. The three lncRNAs antisense or adjacent to *arpc5b, hmcn1, foxp2*, and *cep85l* were also strongly expressed in the epicardium and endocardium. The expression of *lnc-si:dkey-74I1.3* was highest in cardiomyocytes and lowest in the epicardium, a profile that is opposite to the expression distribution of *postnb*. The inverse expression patterns suggest that *lnc-si:dkey-74l1.3* may negatively regulate *postnb* expression in the epicardium and cardiomyocytes. Both genes were expressed at approximately equal levels in the endocardium. The expression of *lnc-si:ch211-108p6.6* was not detected in the three major cardiac tissues. Quantification of *cmlc2*, *tcf21*, and *fli1* expression levels in sorted cells confirm the enrichment of cardiac differentiation markers in specific cell populations after FACS (Fig. 4d). Thus, the expression and distribution analyses of lncRNAs suggest that these noncoding RNAs regulate myocardium regeneration by stimulating epicardium and endocardium activity to create an instructive injury microenvironment.

### Identification of novel lncRNAs expressed during early stages of zebrafish heart regeneration

**Fig. 5:**
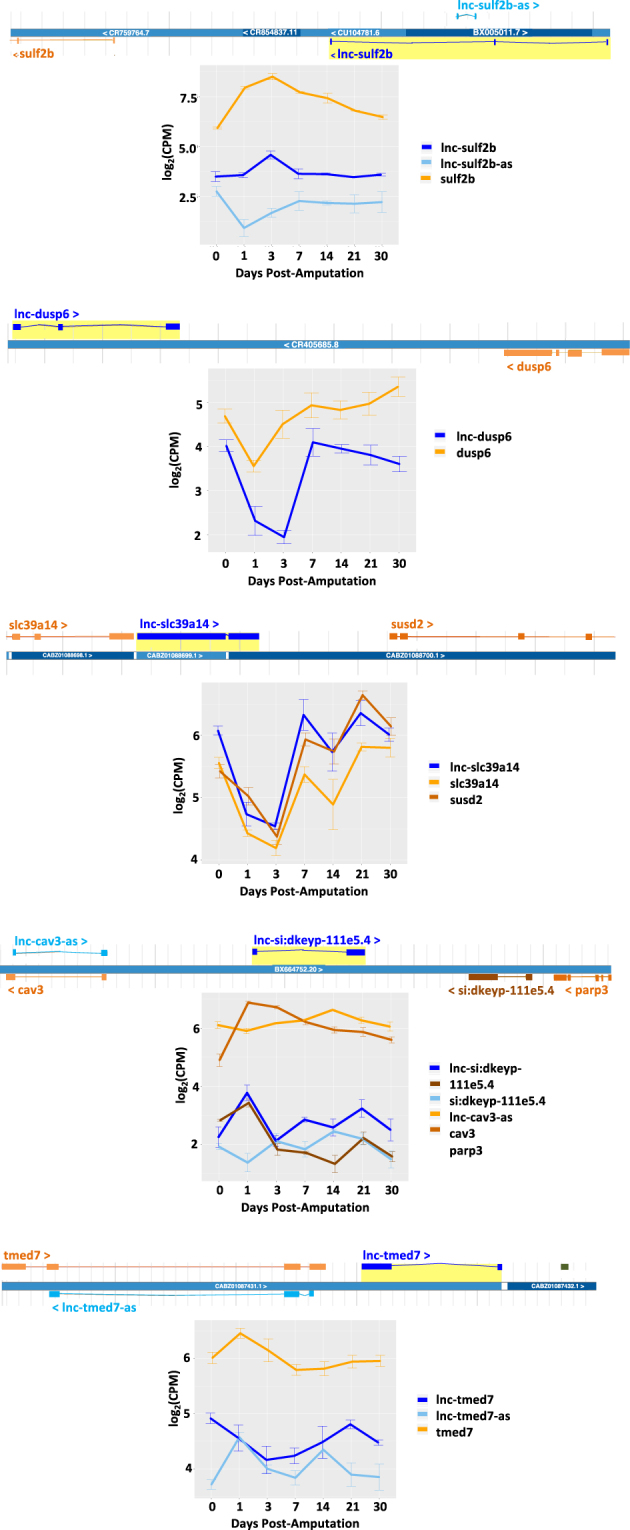
Unique lncRNAs and associated target genes during zebrafish and mouse heart regeneration. Expression of select zebrafish novel lncRNAs adjacent to protein-coding genes and corresponding protein-coding genes that were differentially expressed at 1 and/or 3 dpa relative to 0 dpa. Error bars indicate standard error of the mean

We applied an analysis workflow to identify potential novel lncRNAs expressed during heart regeneration that had not been previously annotated (Supplementary Fig. S4a). After modeling transcripts based on aligned reads using StringTie,^71^ we identified a set of 3741 genes not annotated by Ensembl and identified a subset of 2820 putative noncoding genes. A set of 743 putative novel lncRNAs had multiple exons and a length greater than 200 bp and 429 were differentially expressed at early (1 and 3 dpa) or later time points compared to uninjured tissues (Supplementary Fig. S4b, Supplementary Table S10 and S11). Examination of the 119 putative novel lncRNAs unique to 1 dpa and/or 3 dpa revealed 28 that were not highly repetitive and lacked strong protein alignments. Five of these candidates were antisense or adjacent to differentially expressed protein-coding genes with previously described roles in cell proliferation or heart development (Fig. 5). A cell growth regulator, sulfatase 2b (*sulf2b*), that regulates WNT signaling^72^ was proximal to a possible novel lncRNA, *lnc-sulf2b*, and antisense to another potential novel lncRNA, *lnc-sulf2b-as*. Expression of *sulf2b* was increased during both heart regeneration as well as a previous study of zebrafish caudal fin regeneration.^73^ The putative novel lncRNA, *lnc-dusp6*, was proximal to dual specificity phosphatase 6 (*dusp6*), and both genes were downregulated during early time points. *Dusp6* mutant mice had increased numbers of cardiomyocytes and heart weight.^74^ *Lnc-susd2* was proximal to sushi domain containing 2 (*susd2*), a receptor shown to interact with galectin-1 to inhibit cellular proliferation in studies of the human homolog.^75^ *Lnc-parp3* was proximal to poly(ADP-ribose) polymerase 3 (*parp3*). The mouse homolog, *parp3*, has been associated with cell cycle progression through DNA break repair and histone modification.^76^ *Lnc-parp3* was also distal to caveolin 3 (*cav3*), a component of myocyte caveolae involved in natriuretic peptide signaling and cardiac hypertrophy in a mouse overexpression model,^77^ but *cav3* was not differentially expressed. Finally, *lnc-tmed7* was distal to transmembrane p24 trafficking protein 7 (*tmed7*), and proximal to *mir7a-3*, but mature Mir-7-P4 was not differentially expressed (Fig. 5). The proximity of these five putative novel lncRNAs to differentially expressed protein-coding genes make them strong candidate regulators of heart regeneration.

### Comparative analysis of zebrafish and neonatal mouse heart regeneration

The ability to perform cross-species comparisons is necessary to identify a conserved regulatory circuit for regeneration of specific tissues. In contrast to other regeneration databases, RegenDbase represents orthologous genes, thereby enabling comparisons among organisms. Here, we compared a previously published RNA-Seq dataset from resected neonatal mouse hearts^78^ with our own profiling data from zebrafish hearts at comparable time points during regeneration. Neonatal murine hearts have been shown to fully regenerate missing heart muscle in a manner analogous to the adult zebrafish heart.^79^ For the neonatal mouse study, we used the expression data from the in vivo study so that we could directly compare regeneration in both models. We reanalyzed the RNA-Seq data from this study and found 215 differentially expressed genes from the 1 dpa sham vs. 1 dpa resection comparison and the 29 genes from the 7 dpa sham vs. 7 dpa resection comparison (Supplementary Table S12). A total of 68 ortholog groups were differentially expressed in at least one contrast in both organisms (Supplementary Table S13). A subset of those ortholog groups that are involved with several biological processes including the immune response, ion transport along with TGFβ, NFκB, and integrin signaling (Supplementary Table S14). NFκB signaling has recently been demonstrated to be required for zebrafish heart regeneration.^80^ In that study, *nfkbiaa* was shown to be highly upregulated during regeneration. Two of the genes, *mpeg1* and *marco*, are associated with macrophages suggesting a common role of the immune system in regeneration, consistent with studies in the neonatal mouse.^78^ Six of these genes, *bub1, casc5, cenpe, cenph, nuf2*, and *spc25*, are involved in regulating cell cycling. These genes interact during the spindle assembly checkpoint during cell division.^81^

Looking beyond in vivo comparisons in the neonatal mouse study, we also examined the expression of *il13* which has been shown to induce cardiomyocyte proliferation in vitro by regulating proliferation through *Stat3* and *Stat6*.^78^ We observed that zebrafish *il13* was upregulated by 11.14-fold at 3 dpa, and *stat3* was upregulated by 1.74-fold at 1 dpa (Supplementary Table S12). Taken together, the striking similarity between zebrafish and mouse heart regeneration suggest common mechanisms and indicate zebrafish studies are highly informative for understanding mammalian tissue repair and regeneration.

**Fig. 6:**
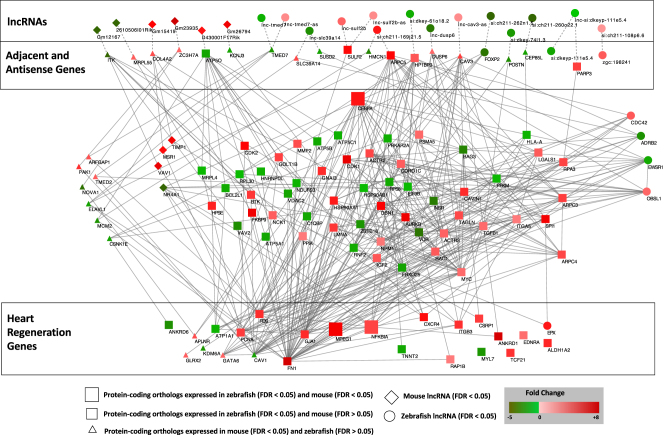
Comparative model of lncRNA-regulation of heart regeneration. Known and novel zebrafish lncRNAs adjacent or antisense to protein-coding genes (top) were connected to a set of previously characterized genes required for heart regeneration in the zebrafish (bottom). Gene regulatory and/or protein–protein interactions from Ingenuity Pathways Analysis were used to find a minimal set of homologous genes that connect the adjacent/antisense genes to the regeneration genes. Genes differentially expressed in the zebrafish (0 vs. 3 dpa) and/or mouse (1 day sham vs. resection) are shown using different shapes and colored by fold change from the zebrafish or mouse (see legend)

To determine the interaction between lncRNAs with heart regeneration genes, we conducted a String analysis integrating differently regulated lncRNAs from the early stages of adult zebrafish and neonatal mouse heart regeneration. In the neonatal mouse heart, 12 lncRNAs were differentially expressed at 1 dpa and two lncRNAs were differentially expressed at 7 dpa (Fig. 6). Three of these 12 lncRNAs are antisense to protein-coding genes. *Lnc*-*Gm15419* expression is upregulated and oriented antisense to the extracellular matrix gene, collagen 4a2 (*Col4a2*). Likewise, *Lnc*-*Gm26794* is also upregulated, but positioned antisense to the potassium channel, *Kcnj3*. A third lncRNA, *lnc-Gm12167*, was downregulated and is antisense to interleukin 2 inducible T-cell kinase, *Itk*. These interaction analyses suggest that a subset of lncRNA-adjacent mRNA units provides an additional layer of regulatory control on expression of conserved heart regeneration genes (Fig. 6). LncRNAs are a relatively new group of gene expression regulators and defining their functional roles during heart regeneration in both regeneration competent and incompetent research systems is a critical next step toward understanding the regulatory complexity of heart regeneration.

## DISCUSSION

We designed and built RegenDbase to facilitate the comparison of diverse regenerative models with a unique focus on noncoding RNAs, critical regulators of regeneration signaling pathways at multiple levels of gene expression. These comparisons may be performed among models with high regenerative capacity or between models with varying capacity. This resource represents gene expression datasets from multiple model systems relevant to regeneration. Our large RNA-sequencing study of zebrafish heart regeneration presented in this study serves as a foundation for comparative analyses to identify noncoding and protein encoding transcripts. The RegenDbase web user interface facilitates the rapid identification of genes differentially expressed within one experiment and across multiple experiments. These comparisons may also be conducted within or across organisms.

Previously, several developmental pathways have been shown to be reactivated following injury to orchestrate complex tissue regeneration.^73,82–84^ Protein-coding genes that make up developmental pathways are highly conserved across vertebrates and are part of the genetic arsenal that coordinate animal development.^85^ Yet, despite the evolutionary conservation of these circuits, the capacity to regenerate tissues is highly variable, suggesting additional layers of regulatory control of gene activation are key to inducing regeneration circuits.

Noncoding RNAs provide two additional layers of regulatory complexity that determine both spatial and relative intensity of developmental pathway activation (Supplementary Fig. S5). First, lncRNAs function in the nucleus to epigenetically regulate target genes that can positively or negatively influence gene expression. Second, noncoding RNAs also can function in the cytoplasm to regulate gene expression post-transcriptionally. In this second layer of genetic complexity, miRNAs negatively regulate target mRNAs, and lncRNAs positively and negatively regulate target gene expression.

RegenDbase facilitates the understanding of miRNA regulation by identifying sets of differentially expressed genes that are predicted to be targeted by miRNAs. As multiple miRNAs can regulate the same gene in a combinatorial fashion, RegenDbase also enables miRNA target searches to identify cases where up to three different miRNAs target the same gene. Using RegenDbase, we have demonstrated how to identify aspects of heart regeneration that are unique to the zebrafish and are conserved with neonatal mouse heart regeneration. The utility of RegenDbase will grow as more datasets from more models are added.

In contrast to miRNAs, lncRNAs are not highly conserved. It is interesting to speculate that a common set of lncRNAs may control common regeneration pathways for multiple tissues, or that different sets of noncoding RNAs are used to modulate regenerative signaling cascades for individual tissues. Further studies of noncoding RNAs in vertebrates with varying regenerative capacities will help define their roles and test the extent to which they do regulate regenerative capacity. Nonetheless, the newly identified noncoding RNAs provide new insight into the complexity of gene regulatory networks that govern regeneration.

RegenDbase has multiple additional search functions that support the development of novel hypotheses for regenerative biology (Supplementary Fig. S1). In this work, we have primarily focused on finding genes that were commonly differentially expressed between zebrafish and mouse. However, RegenDbase supports analysis of genes that are not commonly differentially expressed. For example, the regenerative potential of the neonatal mouse heart is reduced after 7 days. Genes that modulate regeneration may be identified through the comparison of genes that change during postnatal development and those associated with regeneration. In a similar fashion, comparisons of wound healing across organisms with high regenerative capacities to those with low capacities may identify genes that modulate these capacities. While this current release of RegenDbase is focused on zebrafish, axolotl, and mouse data, it is noteworthy to mention that we have established pipelines to integrate future datasets from *C. elegans*, human tissues, and other model organisms to further refine regenerative circuits through comparative analyses.

Additionally, search functionality and more datasets will enhance the system. Studies of inhibited or enhanced regeneration provide valuable insight into genetic mechanisms. Additional data on genomic regulatory features, such as chromatin state and methylation patterns, provide new opportunities to understand these mechanisms. Improvements in single-cell technologies will make it easier to study the contributions of different cell types on regeneration. We also plan to enhance comparative analysis capabilities to facilitate meta-analysis across experiments conducted using different technologies with varying sensitivity and representation of genes. When these experiments are conducted, resources like RegenDbase are primed to integrate, search, and visualize these data to form testable hypotheses for functional studies.

## Methods

### Data collection, relational database, and web interface

Published datasets were loaded in the relational database and checked for any errors related to gene annotation. RegenDbase integrates datasets from 15 experiments and uses 24 external data resources that provide lists of genes and annotations of those genes (see https://regendbase.org/data for list). These resources include genes and transcripts from Ensembl (version 86) and miRBase (version 21), orthologs from OrthoDB (version 9), proteins from UniProt and annotations from the GO and NCBI BioSystems for pathways. miRNAs from miRBase and 3p UTRs from Ensembl were used to make miRNA-binding sites predictions. These data were loaded into the relational database where we have implemented a robust data model (Supplementary Fig. S1). We have designed the system to update these data on a regular basis to take advantage of the latest annotation of genes and genomes.

Central to the RegenDbase data model are RNA segments that represent transcripts and microarray probes. These RNA segments were used to represent genes, assign expression data, and represent miRNA target predictions. Genes may have one or more RNA segments that were assigned any number of annotations. Representing homologous genes, such as orthologs, requires relating genes and their RNA segments to one another across organisms. For protein-coding genes, we represented groups of orthologous genes from OrthoDB (version 9). For axolotl, we first mapped of microarray probe set target sequences to an axolotl transcriptome assembly^14^ using NCBI BLAST blastn to return the first alignment with an expectation value less than 1.0 × 10^−5^. The transcriptome contig that best represented the target sequence was then aligned to the human UniProt protein sequences using NCBI BLAST blastx. The first UniProt alignment returned by BLAST with an expectation value less than 1.0 × 10^−5^ was used to assign to the microarray probe set to the axolotl gene record based on human UniProt mapping to the OrthoDB homolog group. For orthologous miRNAs, we used miRBase (version 21) mapping of miRNAs to RFAM miRNA families. Gene expression data were represented in the data model as experiments, with samples organized into sample groups. Normalized expression values were assigned to RNA segments for each sample. Precomputed comparisons of RNA segment expression between sample groups from the analysis pipelines were represented as fold change and adjusted *p*-values. All these data can then be quickly searched using the web interface. RegenDbase was implemented using Java and the PostgreSQL relational database management system (Supplementary Fig. S1). Source code was maintained using Subversion (http://subversion.apache.org).

### Animal husbandry and tissue collection

All animal procedures were performed, with IACUC approval, in the Animal Core Facility at MDI Biological Laboratory. Female and male adult wild-type Ekkwill (EK) and transgenic zebrafish of 9–12 months of age were anesthetized by immersion in 1:1000 dilution of 2-phenoxyethanol and subjected to ventricular resection. Approximately 20% of the apex was removed, as previously described.^5,12^ Whole ventricles were collected at 0, 1, 3, 7, 14, 21, and 30 days post-amputation (dpa) for RNA extraction.

### RNA preparation and sequencing

Total RNA samples were extracted from triplicate tissue samples using the Zymo Direct-zol RNA microprep kit (Zymo Research Corp., Irvine, CA). Each replicate consisted of 4–6 ventricles. Indexed small RNA libraries and strand-specific polyA+ selected mRNA libraries were prepared and paired-end sequenced on an Illumina HiSeq2500 at the HudsonAlpha Institute for Biotechnology following the manufacturer’s protocols. Sequence data are available in the Gene Expression Omnibus (GSE106884) and Short Read Archive (SRP124960).

### FACS and gene expression

Resected transgenic adult zebrafish ventricles were extracted and dissociated in accordance to previously described methods.^86^ Isolation of fluorescently marked cells from *Tg(tcf21:Dsred), Tg(cmlc2:GFP)*, and *Tg(fli1a:GFP)* was performed on a FACSAria II instrument (BD Biosciences) at the Jackson Laboratory (Bar Harbor, ME). Cells were sorted directly into Trizol LS and total RNA was isolated using Zymo Direct-zol RNA microprep kit, as suggested by the manufacturer (Zymo Research Corp., Irvine, CA). Total RNA was amplified with the Nugen Ovation Pico WTA System and cDNA was synthesized using NEB ProtoScript II First Strand cDNA kit, in accordance to the manufacturer’s protocol (NEB, Ipswich, MA). Real-time qPCR expression studies were performed with SYBR Green (Agilent) detection and specific primers for each gene (Supplementary Table S15).

### Sequence annotation and RNA-Seq data analysis

Following sequence read quality control diagnostic analyses using FastQC version 0.11.2 (http://www.bioinformatics.babraham.ac.uk/projects/fastqc/), small RNA-Seq reads were adapter clipped and mapped to annotated zebrafish miRNAs from miRGeneDB version 1.1 (ref. ^87^) using miRExpress version 2.0.^88^ Sequence tag counts for annotated mature miRNAs from miRGeneDB were analyzed using the R statistical computing environment (http://r-project.org) version 3.2.1 and R/edgeR version 3.2.1.^89^ mRNA-Seq read data were also subjected to quality control diagnostic analyses using FastQC. Reads were trimmed using Trimmomatic version 0.32.^90^ Trimmed paired-end reads were aligned to the Ensembl annotated transcriptome (version 79)^91^ using RSEM version 1.2.25 (ref. ^92^) and Bowtie 1.1.2.^93^ Read counts expressed as transcripts per million were analyzed using R/edgeR. All heatmaps were generated using the R/ComplexHeatmap version 1.0.0 package. GO enrichment analyses were performed using GOrilla^50^ where all genes expressed at each time point were used as background. Zebrafish genes annotated to specific GO terms were obtained using AmiGO.^23^ miRNA-binding sites were predicted using miRanda version 3.3a^94^ using annotated 3p UTRs from Ensembl version 79 and mature miRNA sequences from miRGeneDB version 1.1. Novel lncRNAs were identified by aligning the RNA-Seq reads to the zebrafish genome using HISAT2 version 2.0.5,^95^ generating gene models using StringTie version 1.3.1c,^71^ comparing transcripts to Ensembl version 79 annotation using gffcompare. Novel genes were filtered for repetitive sequences using RepeatMasker and coding potential by CPAT version 1.2.1.^96^

### Disclaimer

The contents of this paper are solely the responsibility of the authors and do not necessarily represent the official views of the NIH or other funding agencies

### Data availability

All expression analysis data have been deposited at the NCBI Gene Expression Omnibus (GSE106884) and the NCBI Short Read Archive (SRP124960).

### Code availability

The web interface to RegenDbase is freely accessible at https://regendbase.org.

## Electronic supplementary material

Supplemental Figure Legends

Supplementary Figure S1

Supplementary Figure S2

Supplementary Figure S3

Supplementary Figure S4

Supplementary Figure S5

Supplementary Table S1

Supplementary Table S2

Supplementary Table S3

Supplementary Table S4

Supplementary Table S5

Supplementary Table S6

Supplementary Table S7

Supplementary Table S8

Supplementary Table S9

Supplementary Table S10

Supplementary Table S11

Supplementary Table S12

Supplementary Table S13

Supplementary Table S14

Supplementary Table S15
