# Supplementary material for: RegenDbase: a comparative database of noncoding RNA regulation of tissue regeneration circuits across multiple taxa
Source: NPJ Regen Med. 2018 May 29;3:10. doi: 10.1038/s41536-018-0049-0 (PMC5973935; doi:10.1038/s41536-018-0049-0)
Supplement: Supplementary file 1 — Supplemental Figure Legends [file 41536_2018_49_MOESM1_ESM.docx]

**Supplementary Figure S1**: **RegenDbase data model represented as relational database tables in an entity relationship diagram.** RegenDbase represents genes as a set of transcripts called RNA segments (blue table labeled “rna_segment”). These RNA segments are used to represent genes, assign expression data and represent miRNA target predictions. Genes may have one or more RNA segment by grouping them together using the “gene_rna_segment” table. Orthologous genes are related to one another in the “gene_homology” table. Annotations for genes, such as Gene Ontology annotations, are represented using the set of green colored tables. These tables are needed to store and traverse directed acyclic graphs that are encountered with ontologies where a term can have multiple parent terms. Protein-protein and gene regulatory relationships among genes are represented in the “gene_gene_reference” table. Gene expression data are represented as experiments with samples organized into sample groups using the set of tables that are colored yellow. The “experiment” table has one or more sample groups (“sample_group” table) that have one or more samples (“sample” table) that have one or more treatments (“sample_treatment” table). The normalized expression values for a RNA segment in a sample are stored in the “rna_segment_sample” table. Pre-computed comparisons of RNA segment expression between sample groups from the analysis pipelines represent fold change and adjusted p-values and are stored in the “sample_group_diff” table. MicroRNA target predictions are represented in the blue “mirna_target” table. The gray tables are used to support accession numbers and hyperlinks to external resources. Experiments and genes have references that are represented in the “reference” table that is colored red.

**Supplementary Figure S2**. **Clustering of differentially expressed mRNAs during zebrafish heart regeneration**. **a)** Heatmap of normalized mRNA expression across timepoints clustered by the timepoint with maximal expression (left panel) and minimal expression (right panel) for all 16,148 genes expressed in each sample. Normalized expression is colored to indicate not detected (black) and then a range from low (blue) to high (green). **b)** Number of upregulated (red) and downregulated (green) genes (FDR < 0.05) for 1, 3, 7, 14, 21 and 30 dpa relative to 0 dpa (left y-axis). Proportion of genes annotated as immune system process (GO:0002376; red), metabolic process (GO:0008152; blue), developmental process (GO:0032502; orange), heart development (GO:0007507; yellow), cell migration (GO:0016477; green) and cell proliferation (GO:0008283; pink) (right y-axis).

**Supplementary Figure S3.** ***tagln* and *tob1a* genes are dynamically regulated during zebrafish heart regeneration.** Expression of *tagln* and *tob1*a at 0, 1, 3, 7, 14, 21 and 30 dpa in log_2_-tranformed counts per million units. Error bars indicate standard error of the mean.

**Supplementary Figure S4.** **Pipeline for identifying novel lncRNA during zebrafish heart regeneration. a)** Novel lncRNA discovery workflow where the set of 42,986 genes modeled by StringTie were filtered to a set of 743 multi-exonic novel non-coding genes. Genes previously annotated by Ensembl were first removed and remaining genes were filtered using RepeatMasker. Next, genes with open reading frames (CPAT < 0.38) were excluded. Finally, single exon genes were excluded. **b)** Comparison of differentially expressed novel lncRNAs at 1 and 3 dpa relative to 0 dpa compared to those differentially expressed at later timepoints.

**Supplementary Figure S5.** **A model for noncoding RNA regulation of regeneration signaling pathways.** Following injury, differential expression of noncoding RNAs modulate the activation and repression of canonical signaling pathways, such as retinoic acid (RA), Wnt, fibroblast growth factor (FGF) and tumor growth factor beta (TGFβ). These signaling pathways are modulated by noncoding RNAs, such as miRNAs and lncRNAs. The signaling pathways modulated by noncoding RNAs govern regenerative processes.

**Supplementary Table S1.** **Functional genomic experiments of regenerative biology in RegenDbase.** Experiment title and reference for each of 15 experiments integrated into RegenDbase.

**Supplementary Table S2**. **miRNA expression during zebrafish heart regeneration.** Table of miRNA expression analysis results for these pairwise sample group comparisons: 0dpa vs. 1dpa, 0dpa vs. 3dpa, 0dpa vs. 7dpa, 0dpa vs. 14dpa, 0dpa vs. 21dpa, and 0dpa vs. 30dpa. Listed for each pairwise sample group comparisons are the log_2_-transformed fold change (e.g., logFC_0dpa_vs_1dpa), average level of log_2_-transformed expression (e.g., logCPM_0dpa_vs_1dpa), log-ratio statistic (e.g., LR_0dpa_vs_1dpa), unadjusted p-value (e.g., PValue_0dpa_vs_1dpa) and false-discovery-rate adjusted p-value (e.g., FDR_0dpa_vs_1dpa). “NA” values indicate the that miRNA was not significantly (FDR > 0.05) differentially expressed in a pairwise sample group comparison.

**Supplementary Table S3**. **Maximal and minimal mRNA expression associated with each stage of zebrafish heart regeneration**. Excel workbook with a total of 14 sheets that list the genes with the highest or lowest expression at each time point sampled (0, 1, 3, 7, 14, 21 and 30 dpa). The first seven of the 14 sheets list the genes with the highest expression at each of the 7 time points. The second group of seven sheets list the genes with the lowest expressed at each of the 7 time points. Each sheet lists the log_2_-transformed CPM values for each sample along with the average for the sample group.

**Supplementary Table S4**. **mRNA expression during zebrafish heart regeneration.** Excel workbook with a total of six sheets that list gene expression analysis results for each of these sample group comparisons: 0dpa vs. 1dpa, 0dpa vs. 3dpa, 0dpa vs. 7dpa, 0dpa vs. 14dpa, 0dpa vs. 21dpa, and 0dpa vs. 30dpa. Each sheet lists the Ensembl gene identifier, log_2_-transformed fold change, log_2_-transformed average level of expression, F-statistic, unadjusted p-value, false-discovery-rate (FDR) adjusted p-value, gene symbol, description and gene type as provided by Ensembl.

**Supplementary Table S5**. **Differentially expressed heart development genes during zebrafish heart regeneration.** Excel workbook with a total of six sheets that list gene expression analysis results for genes annotated to be involved with heart development (GO:0007507) for each of these sample group comparisons: 0dpa vs. 1dpa, 0dpa vs. 3dpa, 0dpa vs. 7dpa, 0dpa vs. 14dpa, 0dpa vs. 21dpa, and 0dpa vs. 30dpa. Each sheet lists the Ensembl gene identifier, log_2_-transformed fold change, log_2_-transformed average level of expression, F-statistic, unadjusted p-value, false-discovery-rate (FDR) adjusted p-value, gene symbol, description and gene type as provided by Ensembl.

**Supplementary Table S6**. **Known regulators of zebrafish heart regeneration.** Excel workbook with a total of six sheets that list gene expression analysis results for genes previously associated with heart regeneration for each of these sample group comparisons: 0dpa vs. 1dpa, 0dpa vs. 3dpa, 0dpa vs. 7dpa, 0dpa vs. 14dpa, 0dpa vs. 21dpa, and 0dpa vs. 30dpa. Each sheet lists the Ensembl gene identifier, log_2_-transformed fold change, log_2_-transformed average level of expression, F-statistic, unadjusted p-value, false-discovery-rate (FDR) adjusted p-value, gene symbol, description and gene type as provided by Ensembl.

**Supplementary Table S7**. **Cellular migration genes differentially expressed during zebrafish heart regeneration.** Excel workbook with a total of six sheets that list gene expression analysis results for genes annotated to be involved with cell migration (GO:001647) for each of these sample group comparisons: 0dpa vs. 1dpa, 0dpa vs. 3dpa, 0dpa vs. 7dpa, 0dpa vs. 14dpa, 0dpa vs. 21dpa, and 0dpa vs. 30dpa. Each sheet lists the Ensembl gene identifier, log_2_-transformed fold change, log_2_-transformed average level of expression, F-statistic, unadjusted p-value, false-discovery-rate (FDR) adjusted p-value, gene symbol, description and gene type as provided by Ensembl.

**Supplementary Table S8**. **Enriched Gene Ontology Biological Process terms during zebrafish heart regeneration**. Excel workbook with a total of six sheets that list enriched Gene Ontology Biological Process terms for each of these sample group comparisons: 0dpa vs. 1dpa, 0dpa vs. 3dpa, 0dpa vs. 7dpa, 0dpa vs. 14dpa, 0dpa vs. 21dpa, and 0dpa vs. 30dpa. Each sheet lists the GO term identifier, GO term name, unadjusted p-value, false-discovery-rate adjusted p-value, number of genes in background (N), number of genes in background annotated to GO term (B), number of target genes (n), number of target genes annotated to GO term (b) and the list of genes as reported by GOrilla with an FDR < 0.05.

**Supplementary Table S9**. **Differentially expressed transcription factors during zebrafish heart regeneration that are predicted direct miR-101 target genes.** Excel workbook with a total of seven sheets. The first sheet lists the six miR-101 target genes that were differentially expressed (FDR < 0.05) and lists the Ensembl gene identifier, log_2_-transformed fold change, log_2_-transformed average level of expression, F-statistic, unadjusted p-value, false-discovery-rate (FDR) adjusted p-value, gene symbol, description and gene type as provided by Ensembl. The other six sheets show predicted miRNA binding sites found in the 3p untranslated regions in transcripts for each gene.

**Supplementary Table S10.** **Expression of 245 putative novel lncRNAs expressed during zebrafish heart regeneration.** Excel workbook with a total of six sheets that list gene expression analysis results for the 245 putative novel lncRNAs (regardless of significance) for each of these sample group comparisons: 0dpa vs. 1dpa, 0dpa vs. 3dpa, 0dpa vs. 7dpa, 0dpa vs. 14dpa, 0dpa vs. 21dpa, and 0dpa vs. 30dpa. Each sheet lists the StringTie gene identifier, log_2_-transformed fold change, log_2_-transformed average level of expression, F-statistic, unadjusted p-value, false-discovery-rate (FDR) adjusted p-value, Ensembl gene ID (if any), StringTie transcript identifiers, number of exons per transcript, gene symbol (if any), chromosome, gene begin coordinate, gene end coordinate and strand.

**Supplementary Table S11**. **Putative novel lncRNAs expressed during zebrafish heart regeneration.** Excel workbook with a total of six sheets that list gene expression analysis results for the putative novel lncRNAs (with a FDR-adjusted p-value < 0.05) for each of these sample group comparisons: 0dpa vs. 1dpa, 0dpa vs. 3dpa, 0dpa vs. 7dpa, 0dpa vs. 14dpa, 0dpa vs. 21dpa, and 0dpa vs. 30dpa. Each sheet lists the StringTie gene identifier, log_2_-transformed fold change, log_2_-transformed average level of expression, F-statistic, unadjusted p-value, false-discovery-rate (FDR) adjusted p-value, Ensembl gene ID (if any), StringTie transcript identifiers, number of exons per transcript, gene symbol (if any), chromosome, gene begin coordinate, gene end coordinate and strand.

**Supplementary Table S12**. **Differentially expressed mouse heart regeneration genes.** Excel workbook with two sheets that list gene expression analysis results for two pairwise sample group comparisons from GSE64403. The first sheet shows the expression analysis results for day 1 post sham neonatal heart ventricles compared to day 1 post resection neonatal heart ventricles. The second sheet shows the expression analysis results for day 7 post sham neonatal heart ventricles compared to day 7 post resection neonatal heart ventricles. Each sheet lists the Ensembl gene identifier, fold change, log_2_-transformed fold change, log_2_-transformed average level of expression, unadjusted p-value, false-discovery-rate (FDR) adjusted p-value, chromosome, gene begin position, gene end position, strand, gene symbol, gene type and description provided by Ensembl.

**Supplementary Table S13**. **Orthologous zebrafish and mouse genes differentially expressed during zebrafish and neonatal mouse heart regeneration**. Orthologous genes are grouped by OrthoDB identifiers listed in the first column. Additional columns list the pairwise sample group comparison, Ensembl gene identifier, fold change, log_2_-transformed fold change, log_2_-transformed average level of expression, unadjusted p-value, false-discovery-rate (FDR) adjusted p-value, gene symbol and description provided by Ensembl.

**Supplementary Table S14**. **Subset of orthologous zebrafish and mouse genes differentially expressed during zebrafish and neonatal mouse heart regeneration.** Orthologous genes are grouped by function listed in the first column. Additional columns list the zebrafish gene symbol, zebrafish pairwise sample group comparison and fold change, mouse gene symbol and mouse pairwise sample group comparison and fold change.

**Supplementary Table S15.** **Primer sequences for real-time qPCR analysis of lncRNAs and protein coding genes.** Forward and reverse oligonucleotide primer sequences for genes shown in Figure 4.
