# Supplementary figures and images for: RegenDbase: a comparative database of noncoding RNA regulation of tissue regeneration circuits across multiple taxa
Source: NPJ Regen Med. 2018 May 29;3:10. doi: 10.1038/s41536-018-0049-0 (PMC5973935; doi:10.1038/s41536-018-0049-0)

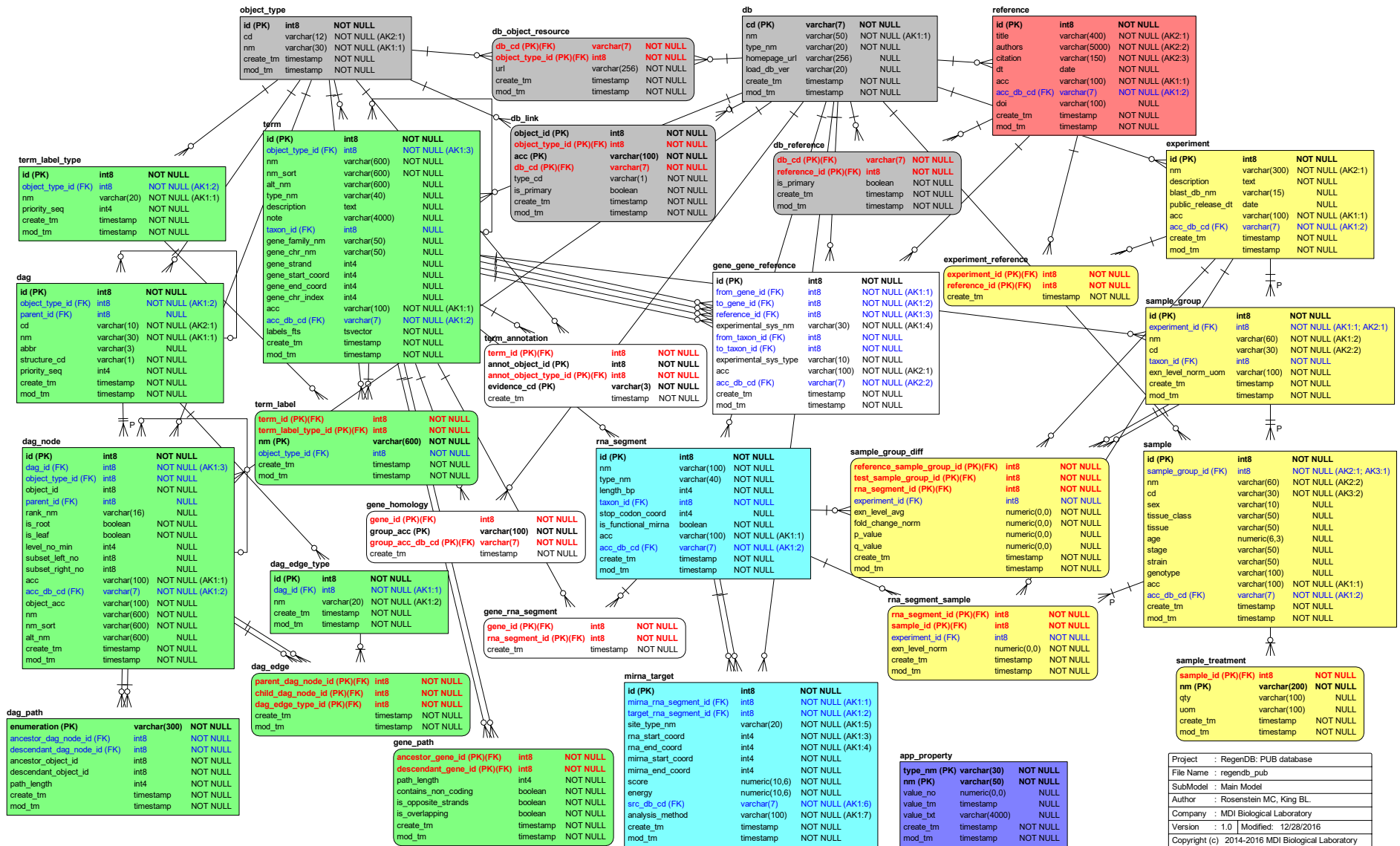

Supplementary Figure S1

Supplement: Supplementary file 2 — Supplementary Figure S1 [file 41536_2018_49_MOESM2_ESM.pdf]

**a**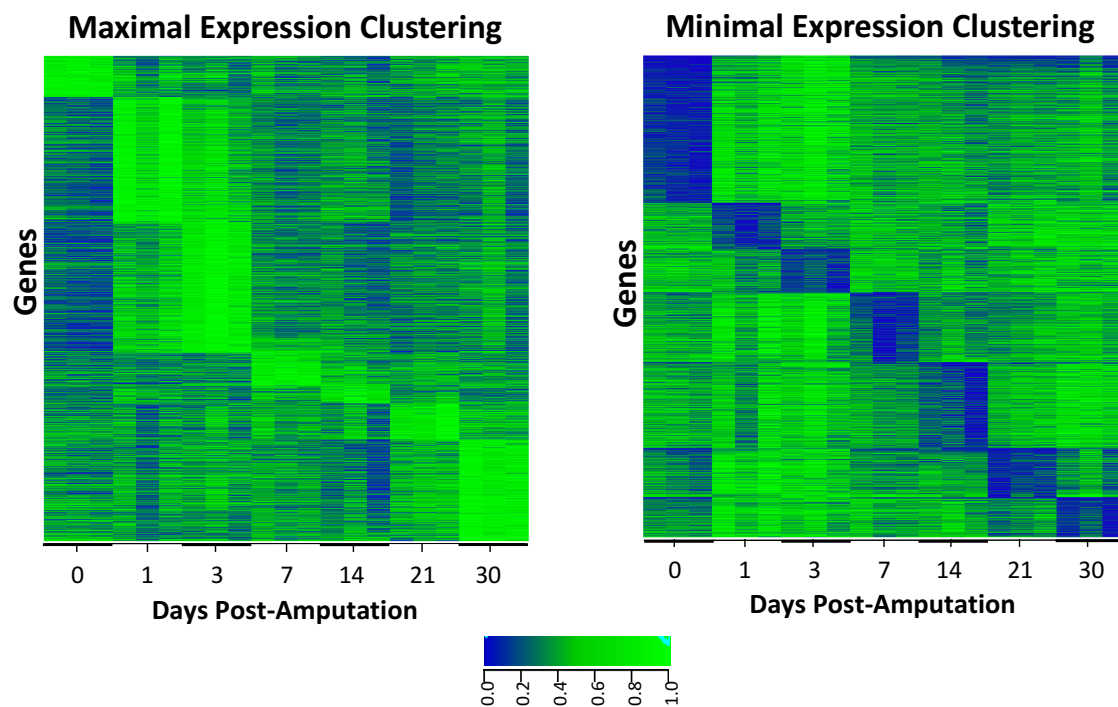**b**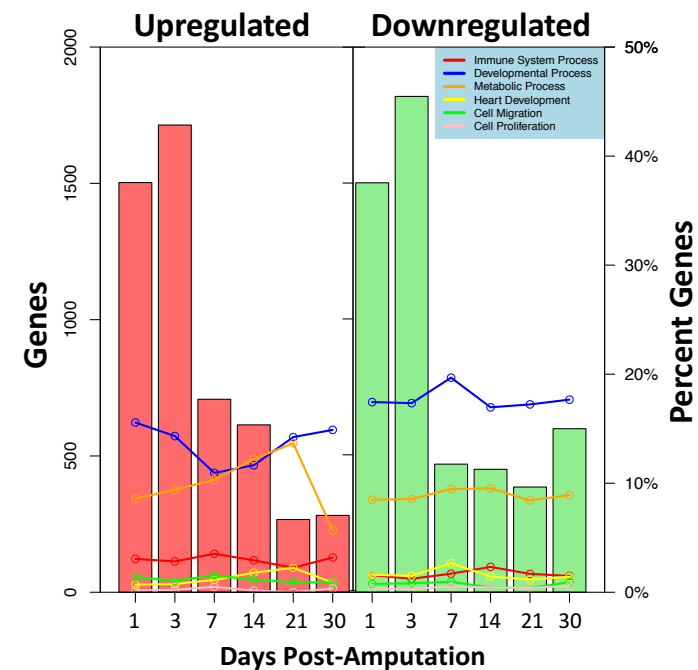**Supplementary Figure S2**

Supplement: Supplementary file 3 — Supplementary Figure S2 [file 41536_2018_49_MOESM3_ESM.pdf]

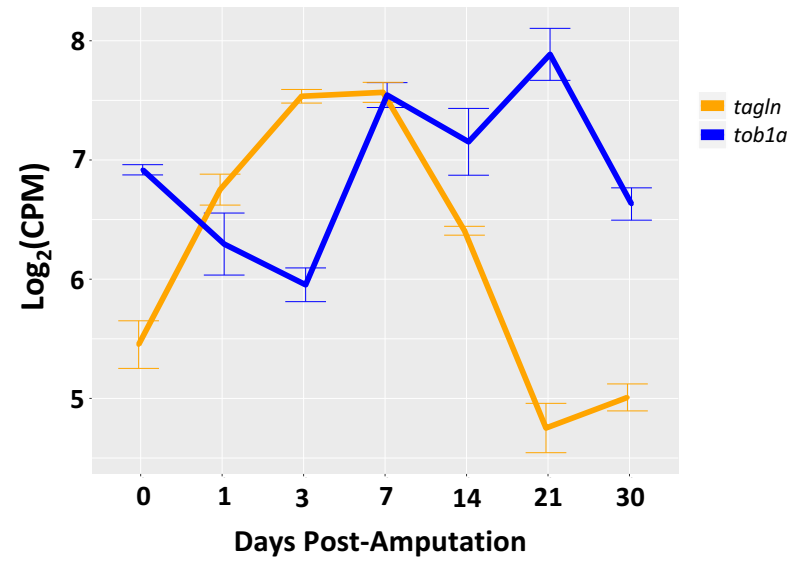

Supplementary Figure S3

Supplement: Supplementary file 4 — Supplementary Figure S3 [file 41536_2018_49_MOESM4_ESM.pdf]

**a**

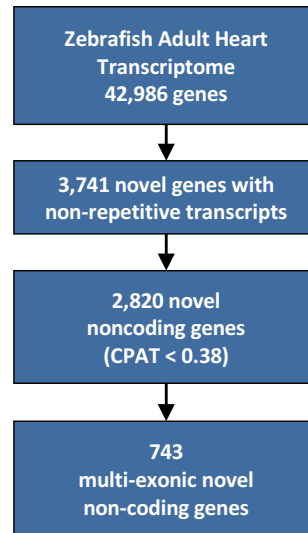

**b**

**LncRNAs in Zebrafish Adult Heart**

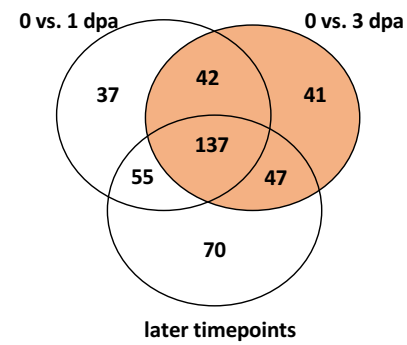

Supplement: Supplementary file 5 — Supplementary Figure S4 [file 41536_2018_49_MOESM5_ESM.pdf]

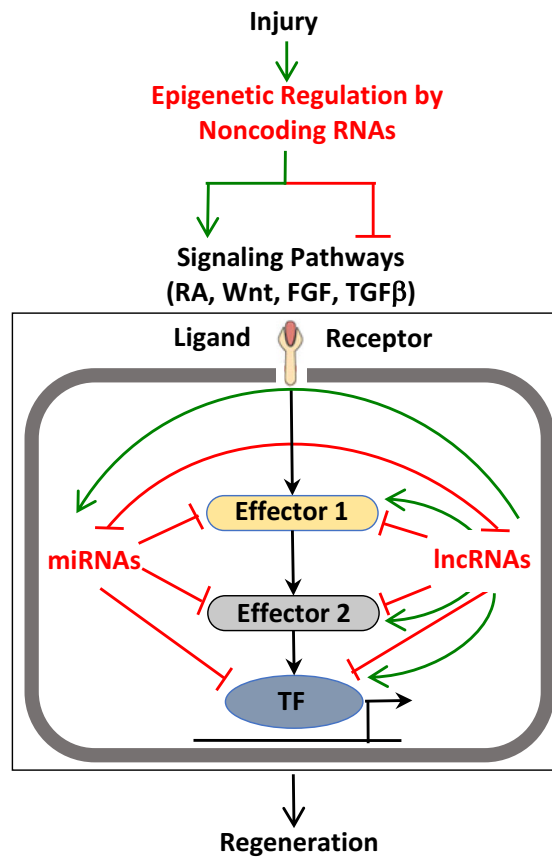

Supplementary Figure S5

Supplement: Supplementary file 6 — Supplementary Figure S5 [file 41536_2018_49_MOESM6_ESM.pdf]
